# The hepcidin concentration decreases in hypothyroid patients with Hashimoto’s thyroiditis following restoration of euthyroidism

**DOI:** 10.1038/s41598-019-52715-3

**Published:** 2019-11-07

**Authors:** Aleksandra Hernik, Ewelina Szczepanek-Parulska, Dorota Filipowicz, Ali Abdolall, Martyna Borowczyk, Elżbieta Wrotkowska, Agata Czarnywojtek, Zbigniew Krasiński, Marek Ruchała

**Affiliations:** 1 Department of Endocrinology, Metabolism and Internal Medicine, Poznan University of Medical Sciences, Poznan, Poland; 2 Department of Vascular and Endovascular Surgery, Angiology and Phlebology, Poznan University of Medical Sciences, Poznan, Poland

**Keywords:** Thyroid diseases, Diagnostic markers

## Abstract

The purpose of the study was to measure the hepcidin concentration and evaluate Fe homeostasis indices in a prospective study on patients with newly diagnosed hypothyroidism in the course of Hashimoto’s thyroiditis (HT) and following successful therapy. The prospective observational study consisted of 34 patients. The clinical evaluation and laboratory tests were performed at diagnosis (T0) and after restoration of euthyreosis 12 weeks later (T1). The median level of hepcidin was significantly lower (p = 0.002) after recovery (7.7 [6.2–13.0] ng/mL) than that before treatment (17.4 [7.6–20.4] ng/mL), while creatinine (p = 0.011) and GFR (p < 0.001) significantly improved after euthyroidism was achieved. A positive correlation was observed between hepcidin and fT3 (p = 0.033, r = 0.465) at T0. In the females, the level of hepcidin positively correlated with ferritin concentration before (p < 0.001, r = 0.928) and after treatment (p < 0.001, r = 0.835). A statistically significant difference was observed in RDW-CV (red blood cell distribution width - coefficient of variation) between the hypothyroid and euthyroid states. In conclusion, a decrease in hepcidin concentration during the transition from the hypothyroid state to euthyroidism in patients with HT is associated with the observed dynamics in iron homeostasis, mainly reflected by improvement in RDW-CV and significant correlations between ferritin and hepcidin as well as between hepcidin and fT3.

## Introduction

The occurrence of anaemia in hypothyroidism is a common finding^1^. It coexists in up to 20–60% of hypothyroid patients^2^. Moreover, thyroid dysfunction is one of the most frequently diagnosed occult causes of recurrent or resistant anaemia, while restoration of euthyroidism improves haematological parameters^3^. On the other hand, indicators of anaemia detected during laboratory tests might be the first manifestation of thyroid disease in asymptomatic patients. Among hypothyroid patients, the most common is normocytic anaemia, followed by macrocytic and the least common, microcytic^4^.

In hypothyroidism, normocytic anaemia may be a manifestation of a general decrease in basal metabolism that results in reduced tissue oxygen demand^5,6^. Macrocytic anaemia in Hashimoto’s thyroiditis (HT) is frequently due to other autoimmune comorbidities, with pernicious anaemia being diagnosed in up to 10% of HT patients. Other causes of macrocytic anaemia include celiac disease leading to vitamin B12 and folate deficiencies, rheumatological disorders, and rarely by autoimmune haemolytic or Evans syndrome^1^. Thyroid dysfunction constitutes one of the most important causes of macrocytic anaemia next to alcoholism and liver and bone marrow disorders, whereas among macrocytic patients without anaemia, hypothyroidism is a dominant etiological factor^7^. Microcytic anaemia in the course of hypothyroidism is mainly a result of iron (Fe) deficiency that occurs due to impoverished nutrition or malabsorption^8^. It can also be a result of blood loss as an effect of menorrhagia in hypothyroid women. Additionally, microcytic anaemia may also be a result of chronic inflammation due to proinflammatory cytokine activity.

It is known that thyroid hormones stimulate erythropoiesis, directly acting on bone marrow and enhancing erythropoietin (EPO) renal synthesis. Moreover, Fe, as a constituent of thyroid peroxidase, is essential in the process of thyroid hormone synthesis^9,10^. However, the exact patho-mechanism and dominant reason for the frequent coexistence of Fe homeostasis disturbances in HT remain unclear.

Hepcidin is a liver-derived 25-amino acid peptide and is an acute-phase reactant that was first identified in 2000^11^. Two inflammatory signalling pathways regulate the transcription of hepcidin: the Stat3 pathway, which is triggered by interleukin-6 (IL-6) and other inflammatory cytokines, and the Bone Morphogenic Protein (BMP)-derived Smad1/5/8 pathway, which is activated by Activin B binding to the BMP receptor^12^. It also plays a crucial role in iron balance. Within enterocytes, spleen macrophages, and the liver, hepcidin forms a complex with ferroportin and is subsequently degraded. This process consequently prevents iron transfer from tissues to the blood plasma and can lead to hypoferraemia of inflammation^13^. In several diseases, hepcidin overexpression leads to iron-restricted anaemia. Conversely, decreased expression of hepcidin leads to iron overload conditions such as haemochromatosis, β-thalassaemia or congenital dyserythropoietic states. Since hepcidin has been identified as a possible contributor in other autoimmune diseases, it should also be considered as one of the factors increasing the risk of anaemia in the course of HT^14^. We hypothesized that changes in hepcidin levels may be responsible for or at least reflect the disturbances in iron homeostasis in patients with HT during hypothyroidism and after euthyroidism is restored. Thus, the aim of our study was to evaluate the hepcidin concentration and iron homeostasis indices in a prospective study of patients with newly diagnosed hypothyroidism in the course of HT and following therapy.

## Subjects and Methods

We conducted the project at a tertiary reference endocrine centre outpatient clinic. The prospective observational study comprised patients with newly diagnosed hypothyroidism due to HT. We performed clinical evaluation of patients prior to treatment at the time of diagnosis (T0) and following restoration of euthyroidism after treatment (T1). All volunteers were above 18 years of age and underwent endocrinological consultations due to suspicion of hypothyroidism. Only patients with a confirmed diagnosis of hypothyroidism in the course of HT were included. The diagnosis was based on clinical (i.e., weakness, somnolence, cold intolerance, dry skin, weight gain, constipation), laboratory (significant elevation of thyroid-stimulating hormone, decreased free thyroid hormones, positive anti-thyroid antibodies) and ultrasound (decreased, inhomogeneous echogenicity) features. The study excluded patients who suffered from any of the following conditions that occurred during a period of 6 months prior to diagnosis: haemolysis, haemorrhage, surgical therapy, symptomatic anaemia, dietary supplementation (Fe, vitamin B12, or folic acid), active malignancy, pregnancy, breastfeeding, administration of exogenous hormones (oral contraception, hormonal replacement therapy, chronic corticosteroid therapy, EPO), chronic liver and kidney diseases, acute inflammatory diseases, other autoimmune diseases, and previous therapy for hypothyroidism. Postmenopausal women were not included. Informed consent was obtained from all participants. The experimental protocol is in accordance with the 1964 Helsinki Declaration and its later amendments or comparable ethical standards and was approved by the Poznan University of Medical Sciences Bioethical Committee (approval number: 386/16).

Laboratory tests including measurement of hepcidin levels were performed in all patients at the time of diagnosis (T0) and following restoration of euthyreosis 12 weeks later (T1). Patients underwent treatment with L-thyroxine at a dose adjusted according to age, body weight and initial thyroid hormone levels.

Hepcidin levels were measured using Hepcidin 25 (bioactive) high sensitivity enzyme immunoassay HS ELISA for quantitative *in vitro* diagnostic measurement (DRG Instruments GmbH, Germany). Thyroid gland function was assessed by measuring the following parameters: thyroid-stimulating hormone (TSH), free thyroid hormones (free triiodothyronine - fT3, free thyroxine - fT4), anti-thyroid peroxidase antibodies (aTPO) and anti-thyroglobulin antibodies (aTG). In order to exclude the presence of any acute inflammatory processes and chronic liver or kidney diseases, we measured C-reactive protein (CRP), aminotransferases (ALT, AST), and creatinine by the GFR (glomerular filtration rate). Iron (Fe) homeostasis indices were evaluated by measuring the complete blood count (CBC), serum Fe and ferritin. ALT, AST, Fe, creatinine and CRP biochemical parameters were assessed using a Hitachi Cobas e501 analyser (Roche, Diagnostics, Indianapolis, USA), while the measurements of thyroid-related parameters (TSH, fT3, fT4, aTPO, aTG) and ferritin were performed using a Hitachi Cobas e601 chemiluminescent analyser (Roche, Diagnostics, Indianapolis, USA). We estimated the GFR using the online medical calculator (https://www.mdcalc.com/mdrd-gfr-equation) based on the MDRD (The Modification of Diet in Renal Disease Study) equation. CBC was measured using an automated flow cytometer Sysmex-XN 1000 (Sysmex Europe GmbH, Bornbarch, Germany). Thyroid ultrasound examination was performed using an AIXPLORER system (Supersonic Imagine, Aix en-Provence, France).

### Statistical methods

Statistical analysis of acquired data was performed using STATISTICA software (StatSoft, Tulsa, Oklahoma, USA). All figures presented were prepared with PQStat Software (Poland). Nonparametric tests were applied due to the lack of a normal distribution of all parameters. The Wilcoxon signed-rank test was used to compare two related samples (T0 vs T1). Spearman’s rank correlation coefficient was used to evaluate hepcidin levels and all laboratory parameters measured in this study before (T0) and after recovery (T1). Data are presented as the median and 25–75% interquartile range [IQR]. Parameters that have different reference ranges in men and women were calculated separately, such as red blood cells (RBCs), haematocrit (HCT), haemoglobin (HGB) and ferritin. The level of statistical significance was set at p < 0.05. All data needed to reproduce the results are available from the corresponding author on reasonable request.

## Results

The studied population consisted of 34 consecutive patients diagnosed with overt hypothyroidism in the course of HT at baseline. Thirteen patients had to be removed from the final analysis due to excluding factors. Of the 21 patients who fulfilled the strict inclusion criteria, three were lost during follow-up. A final prospective analysis of 18 patients, including 13 women and 5 men, is presented. The mean age of the patients in the study group was 44.5 ± 16.7 years old.

### Hepcidin and other parameters

The comparison of hepcidin and biochemical parameters at the time of diagnosis of HT (T0) and at follow-up (T1) is presented in Table 1.

**Table 1:** The comparison of biochemical parameters at the time of diagnosis of hypothyroidism in the course of Hashimoto’s thyroiditis (T0) and in euthyroidism at follow-up (T1).

Parameter [unit]	Reference range	HT baseline (T0) Median [25–75%]	HT follow-up (T1) Median [25–75%]	p-value*
**Hepcidin [ng/mL]**	**0.3–47.7**	**17.4** **[7.6–20.4]**	**7.7** **[6.2–13.0]**	**0.002**
Fe [µg/dL]	F:37–145	92 [77–97]	78 [66–99]	NS
	M:59–158	77 [77–92]	90 [87–97]	
Ferritin [ng/mL]	F:13–150	48 [23–72]	48 [24–100]	NS
	M:30–400	179 [163–236]	175 [170–223]	
**TSH [µIU/mL]**	**0.2–4.2**	**65.8** **[33.4–100.0]**	**0.9** **[0.1–1.5]**	**<0.001**
**fT3 [pmol/L]**	**3**.**9–6**.**7**	**2**.**6** **[1**.**9–3**.**1]**	**4**.**9** **[4**.**6–6**.**1]**	<**0**.**001**
**fT4 [pmol/L]**	**11**.**5–21**.**0**	**4**.**7** **[2**.**6–7**.**9]**	**20**.**6** **[18**.**7–24**.**1]**	<**0**.**001**
aTPO [IU/mL]	<34.0	508.0 [171.5–600.0]	481.0 [173.5–600.0]	NS
aTG [IU/mL]	10–115	462.0 [183.5–820.7]	251.0 **[**101.2–1002.5]	NS
ALT [U/L]	10.0–41.0	21.0 [14.7–41.0]	20.5 [11.8–40.0]	NS
AST [U/L]	10.0–37.0	26.0 [18.2–40.2]	22.0 [16.0–29.0]	NS
CRP [mg/L]	<5.0	0.5 [0.3–2.7]	1.5 [0.5–5.3]	NS
**Creatinine [mg/dL]**	**0**.**5–1**.**2**	**1**.**0** **[0**.**8–1**.**1]**	**0**.**7** **[0**.**7–0**.**8]**	**0**.**011**
**GFR [mL/min/1**.**73 m2]**	**>60**	**76**.**2** **[58**.**9–84**.**4]**	**95**.**0** **[83**.**3–108**.**0]**	<**0**.**001**

Data represent the median [IQR] and are analysed by the nonparametric Wilcoxon signed-rank test (n = 18). Abbreviations: NS-non-significant. F: parameters with different reference ranges in men and women; test performed in the female subgroup (n = 13). M: parameters with different reference ranges in men and women; test performed in the male subgroup (n = 5). Fe - iron, TSH - thyroid-stimulating hormone, fT3 - free triiodothyronine, fT4 - free thyroxine, aTPO - anti-thyroid peroxidase antibody, aTG - anti-thyroglobulin antibody, ALT - alanine aminotransferase, AST - aspartate aminotransferase, CRP - C-reactive protein, GFR - glomerular filtration rate by the MDRD equation.

**Figure 1:**
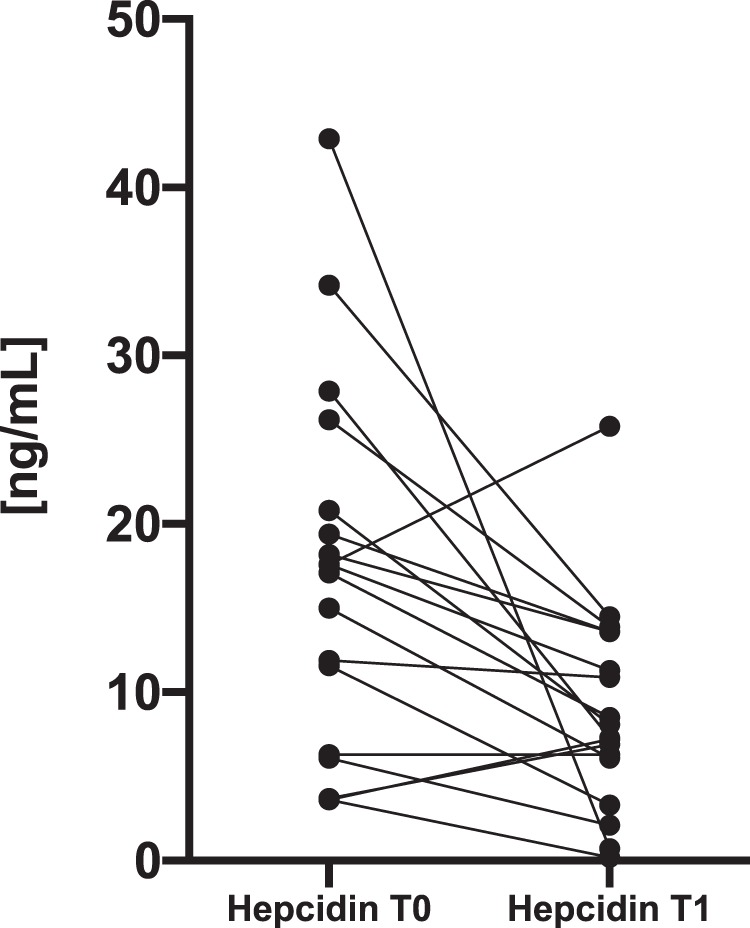
The plot of the individual patients’ hepcidin levels before (T0) and after treatment (T1) of hypothyroidism. Baseline median (17.4 [7.6–20.4] ng/mL) and follow-up (7.7 [6.2–13.0] ng/mL).

**Figure 2:**
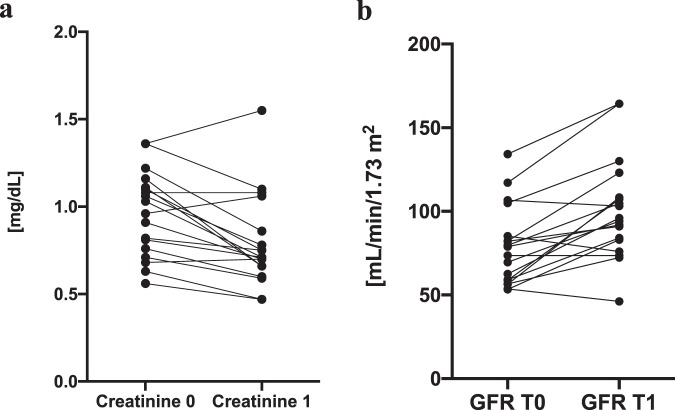
The plots at the time of diagnosis (T0) and after treatment (T1) of hypothyroidism. Data are presented as patients’ individual values. (**a**) creatinine (median [IQR]: T0 = 1.0 [0.8–1.1] mg/dL, median T1 = 0.7 [0.7–0.8] mg/dL). (**b**) GFR (median T0 = 76.2 [58.9–84.4] mL/min/1.73 m^2^, median [IQR] T1 = 95.0 [83.3–108.0] mL/min/1.73 m^2^).

The median [IQR] level of hepcidin was significantly lower (p = 0.002) after recovery (7.7 [6.2–13.0] ng/mL) than that before treatment (17.4 [7.6–20.4] ng/mL) **(**Fig. 1**)**. However, Fe and ferritin remained stable and did not demonstrate significant statistical differences between the hypothyroid and euthyroid states. No statistically significant changes in the levels of anti-thyroid autoantibodies, aminotransferases or CRP were observed in patients following restoration of euthyreosis. Meanwhile, the level of creatinine and the GFR showed statistically significant improvement after euthyroidism was achieved **(**Fig. 2a,b**)**.

**Figure 3:**
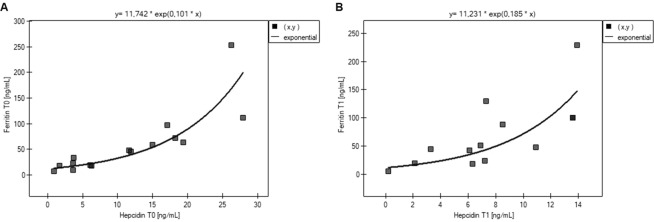
Correlation of the hepcidin and ferritin concentrations in females. (**a**) at the moment of diagnosis of hypothyroidism (r = 0.928, p < 0.001). (**b**) following recovery (r = 0.835, p < 0.001).

A positive Spearman’s correlation was observed between the concentration of hepcidin and fT3 (p = 0.033, r = 0.465) in hypothyroid patients (T0). In females, the level of hepcidin positively correlated with the ferritin concentration before (p < 0.001, r = 0.928) and after treatment (p < 0.001, r = 0.835) **(**Fig. 3a,b**)**.

### The complete blood count

All parameters included in the complete blood count were compared between T0 and T1, and the results are shown in Table 2.

**Table 2:** The comparison of complete blood counts at diagnosis of hypothyroidism (T0) and after euthyroidism was restored at follow-up (T1).

Parameter [unit]	Reference range	HT baseline (T0) Median [25–75%]	HT follow-up (T1) Median [25–75%]	p-value
WBCs [×10^3^/µl]	3.9–11.0	6.7 [6.0–7.8]	6.6 [6.3–8.0]	NS
RBCs [×10^6^/µl]	F:3.5–5.2	4.4 [4.2–4.6]	4.4 [4.2–4.6]	NS
	M:4.2–5.8	4.6 [4.4–4.6]	4.8 [4.5–4.9]	
HGB [g/dL]	F:12.0–15.6	13.2 [12.6–14.0]	13.2 [12.9–13.6]	NS
	M:13.5–17.2	13.9 [13.7–13.9]	15.0 [14.0–15.0]	
HCT [%]	F:33.0–46.0	40.1 [37.4–41.4]	38.4 [38.4–40.4]	NS
	M:39.5–50.5	41.5 **[**41.5–42.3]	44.5 [42.7–44.6]	
**MCV [fl]**	**80**.**0–99**.**0**	**91**.**4** **[89**.**1–94**.**5]**	**90**.**6** **[87**.**5–92**.**6]**	**<0**.**001**
**MCH [pg]**	**27**.**0–33**.**5**	**30**.**9** **[29**.**8–31**.**8]**	**30**.**3** **[29**.**4–31**.**0]**	**0**.**006**
MCHC [g/dL]	31.0–38.0	33.6 [33.0–34.0]	33.6 [33.2–34.0]	NS
**RDW-CV [%]**	**11**.**0–16**.**0**	**13**.**4** **[13**.**1–14**.**1]**	**13**.**1** **[12**.**0–13**.**2]**	**<0**.**001**
PLT × 10^3^/µl	130.0–400.0	242.0 [197.8–277.2]	238.5 [208.2–331.5]	NS
PDW [fL]	9.0–17.0	12.3 [11.1–13,15]	12.4 **[**11.2–12.8]	NS
MPV [fL]	9.0–13.0	10.6 [9.9–11.1]	10.6 [10.0–10.8]	NS
P-LCR [%]	13.0–43.0	30.0 [24.4–32.9]	30.0 [25.4–32.1]	NS

Data represent the median [IQR] and were analysed by the nonparametric Wilcoxon signed-rank test (n = 18). Abbreviations: NS - non-significant. F: parameters with different reference ranges in men and women; test performed in the female subgroup (n = 13). M: parameters with different reference ranges in men and women; test performed in the male subgroup (n = 5). WBCs - white blood cells, RBCs - red blood cells, HGB - haemoglobin, HCT - haematocrit, MCV - mean corpuscular volume, MCH - mean corpuscular haemoglobin, MCHC - mean corpuscular haemoglobin concentration, RDW-CV - red blood cell distribution width - coefficient of variation, PLT - platelets, PDW - platelet distribution width, MPV - mean platelet volume, P-LCR - platelet larger cell ratio.

**Figure 4:**
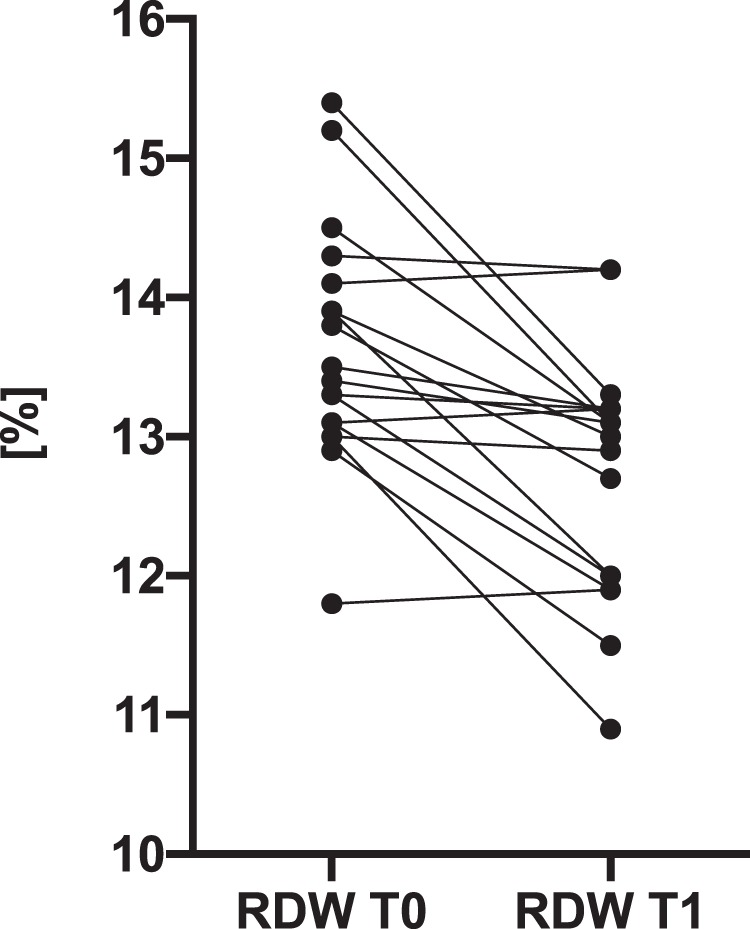
The RDW-CV (red blood cell distribution width - coefficient of variation) plots at the time of diagnosis (T0) of hypothyroidism and after treatment (T1). Data are presented as patients’ individual values (median T0 = 13.4 [13.1–14.1] %; median T1 = 13.1 [12.0–13.2] %).

Despite mean corpuscular volume (MCV), mean corpuscular haemoglobin (MCH) and RDW-CV (red blood cell distribution width - coefficient of variation) being within the normal ranges at baseline (T0) and at follow-up (T1), there were statistically significant differences observed in hypothyroid patients compared to those who were euthyroid **(**Fig. 4**)**. The remaining parameters did not differ significantly.

## Discussion

To the best of our knowledge, our research is the first prospective observational study to compare hepcidin levels and selected haematological parameters reflecting Fe homeostasis in patients with hypothyroidism and HT at diagnosis and following restoration of euthyroidism. The level of hepcidin is regulated by Fe ions in the serum and by numerous stimulating and inhibiting factors. In the majority of situations, a decrease in Fe concentration (e.g., haemolysis or haemorrhage) causes a decrease in hepcidin production. Conversely, states of acute inflammation mediated via IL-6, cancers and chronic and autoimmune diseases lead to an increase in hepcidin levels^12^. According to data provided by the manufacturer that are based on studies conducted on healthy adults, the normal level of hepcidin is 0.3–47.7 (ng/mL). Similar results have been obtained by Bulgarian and Dutch researchers and estimated at 3.1–37.7 (ng/mL)^15^. Kumar *et al*. estimated the median plasma hepcidin in healthy children at approximately 21.9 ng/mL^16^. In our study, we aimed to assess the hepcidin concentration in patients with hypothyroidism. The influence of any other factor, except thyroid status, that may potentially impact hepcidin or Fe homeostasis was excluded. The median level of hepcidin in our group of hypothyroid patients was 17.4 (ng/mL) and dropped to 7.7 ng/mL following therapy.

Thyroid hormones play an important role in haematopoiesis by regulating gene expression and EPO secretion in kidneys, which stimulates proliferation of erythrocyte precursors^5,6,17,18^. Thyroid hormone deficiency is characterized by reduced tissue oxygen requirements. Physiological adaptation leads to decreased EPO secretion, a decrease in the number and proliferative activity of erythroid cells^19^, and promotion of accumulation of mucopolysaccharides in the bone marrow^20^. These processes are reversed following restoration of euthyroidism^1^.

Our data are consistent with the current model in which hypothyroid patients have hypoproliferative anaemia, with low EPO, accompanied by volume contraction, but with a small or no effect on haemoglobin concentration. Once the hypothyroidism is corrected, the erythron and plasma volume expand. This could lower hepcidin through increased EPO generating an erythroid signal (e.g., erythroferrone) and through transiently decreasing serum iron and accumulated iron stores that are used up to make more haemoglobin. However, 12 weeks later, increased iron absorption will have corrected the iron parameters, the erythron is expanded, and a new steady state is reached with higher erythrocyte turnover and lower hepcidin. This may be reflected by the observed increase in RDW-CV concentration, which is a sensitive marker of Fe homeostasis^21,22^.

Hypothyroidism also manifests with myxoedema and decreased plasma volume, which may lead to a relative overestimation of haemoglobin values that conceals an underlying anaemia^19^. Following restoration of euthyroidism and subsequent plasma volume increase, no change in haemoglobin levels or even decreased red blood cell indices might be observed. Thus, in hypothyroid patients, an increased RDW might be the first sign of iron imbalance. In these situations, a more valuable indicator of Fe status may be the ratio between the standard deviation of RBC volumes and MCV, multiplied by 100 (RDW-CV). This might explain the observed changes in RDW-CV values during the transition from hypothyroidism to the euthyroid state in our study, while other red blood cell indices remained unchanged. This parameter has been found to correlate positively with TSH in a healthy population and was suggested by Aktas *et al*. to be a marker for hypothyroidism in women, after excluding patients with Fe-deficient anaemia^21,23^. Clinically, RDW is primarily used to detect microcytic anaemia^24^. Therefore, a higher value of the parameter reflects a greater degree of anisocytosis. Thus, when a decrease in RDW-CW occurs, there is evidence of an improvement in iron balance and better iron supply, enabling the formation of RBCs of normal volume, reflecting more effective erythropoiesis. In our previous study on patients with heart failure and hypothyroidism after radioiodine therapy, there was a strong correlation between TSH levels and RDW^22^. Moreover, RDW has been found to correlate positively with TSH levels in a healthy population and has been suggested by Aktas *et al*. to be a marker for hypothyroidism in females after excluding patients with Fe-deficient anaemia^21,23^. Yu *et al*. also reported an association between RDW and TSH in a large cohort of euthyroid and subclinically hypothyroid patients, where the correlation was strongest for TSH above the normal range^25^. In our study of hypothyroid patients, the RDW-CV levels were within normal ranges. However, there was a statistically significant improvement after restoration of euthyroidism, which may reflect decreased anisocytosis. Meanwhile, no significant differences in Fe, ferritin, haemoglobin, HTC and RBC levels were observed, which is in accordance with previous reports^26^. We did not observe significant changes in the platelet (PLT) count after L-thyroxine introduction, which contradicts previous data from studies in mice suggesting that thyroid hormones may suppress thrombocytopoiesis^27^. While the results of MCV and MCH might be somewhat ambiguous, our results are compatible with Horton *et al*., who observed diminished anisocytosis after L-thyroxine supplementation despite normal baseline and after treatment MCV values^28^.

Hepcidin concentration data in thyroid dysfunction are scarce. Two previous studies have compared hepcidin concentrations in patients with hyperthyroidism before and after successful treatment. The first study, conducted by Lehtihet *et al*., failed to demonstrate significant differences in the serum hepcidin or Fe concentration in hyperthyroidism compared to the euthyroid state, accompanied by a significant rise in ferritin levels^29^. In the second study by Fischili *et al*. evaluating patients with hyperthyroidism due to Graves’ disease, the authors noted that ferritin and hepcidin levels measured only by mass spectrometry and only in the male subgroup showed a statistically significant decrease, also with no changes in Fe levels, while hepcidin measured by the ELISA method did not differ between the groups^30^. In the latest study by our team, we demonstrate that subacute thyroiditis is associated with a significantly higher concentration of hepcidin when compared to values obtained after remission of the disease is achieved^31^. However, in contrast to the present study, patients with subacute thyroiditis present with systemic inflammation, which generates an inflammatory signal mediated by IL-6 that raises hepcidin. This is reflected by very high CRP levels at diagnosis, which are normalized during remission. However, that mechanism was not the case in subjects with HT because the CRP level was within the normal range at diagnosis and during therapy, with no significant changes observed.

Previous studies have also identified a relationship between hepcidin and sex hormones. Administration of testosterone in male subjects resulted in a reduction in hepcidin levels, while an increase in haemoglobin and haematocrit was observed^32^. Moreover, Guo *et al*. have also demonstrated that testosterone suppresses hepcidin transcription by affecting the BMP/Smad signalling pathway^33^. Similarly, an increase in endogenous oestrogen levels in females before *in vitro* fertilization appears to cause hepcidin suppression^29^. Yang *et al*. demonstrated that 17-β-oestradiol suppressed hepcidin gene (HAMP) expression *in vitro* in human cells and therefore increased Fe uptake^34^. According to Galesloot *et al*., the hepcidin concentration depends on the oestrogen status of females, being the lowest in young women and higher following menopause; however, it is independent of age in men^35^. Thus, to ensure that there is no influence of oestrogen status on the hepcidin concentration, our female subgroup consisted entirely of women of premenopausal age with regular menstrual bleeding.

Disturbances in renal functioning in hypothyroid patients have also been reported^36^. In our study, we observed that restoration of euthyroidism was associated with significant improvements in GFR and creatinine. Although renal parameters were within normal ranges in both hypothyroid and euthyroid states, after achieving euthyroidism, GFR and creatinine significantly improved. A prospective observation by den Hollander *et al*. has also reported similar findings^37^.

The majority of previous studies have reported positively correlated hepcidin and ferritin levels in children^38^, critical care unit patients^39^, and patients with respiratory disease^40^ and vasculitis^41^. Our results studying both hypothyroid and healthy subjects are consistent with previous research. Kunireddy *et al*. reported positively correlated hepcidin and ferritin levels in both a healthy female group and another group that included patients with autoimmune diseases such as systemic lupus erythematosus^42^.

Although our study presents novel findings, there are some limitations to be mentioned. Since the sample size was relatively small, our findings need to be verified by future studies on larger populations. We did not compare the hepcidin concentration with a healthy control group, since the study was designed to use the same patients as their own control following successful restoration of normal thyroid function. The strengths of our project were the strict inclusion criteria and careful selection of patients, which allowed us to achieve the situation in which only thyrometabolic status influenced hepcidin and red blood cell indices in the studied patients.

In conclusion, the results of our study suggest that successful treatment of hypothyroid patients with HT and restoration of the euthyroid state is associated with a decrease in hepcidin concentration that follows the observed dynamics of iron homeostasis. This is reflected by an associated improvement in RDW-CV and a significant correlation between the levels of both hepcidin and ferritin and hepcidin and fT3.
